# Draft genome sequences of a strain of *Clostridium neuense* and four *Candidatus* Clostridium species

**DOI:** 10.1128/mra.01274-24

**Published:** 2025-03-20

**Authors:** Yu Chyuan Heng, Jolie Kar Yi Lee, Amber Ching Han Lim, Sandra Kittelmann

**Affiliations:** 1 WIL@NUS Corporate Laboratory, Centre for Translational Medicine, Wilmar International Limited, National University of Singapore 37580, Singapore, Singapore; California State University San Marcos, San Marcos, California, USA

**Keywords:** *Clostridium*, draft genome sequence, *Candidatus* species

## Abstract

We report the draft genomes of five *Clostridium* isolates from soil and agricultural by-products, four of which are proposed as *Candidatus* species. Members of the genus *Clostridium* are of significant industrial interest, and the availability of their genome sequences facilitates the understanding and exploration of their functional potential.

## ANNOUNCEMENT

The taxonomy and systematics of the genus *Clostridium* remain complex and incompletely resolved. As of December 2024, according to the List of Prokaryotic names with Standing in Nomenclature, this genus comprises 162 validly published species (1). However, not all species have available genome sequences, including *Clostridium neuense*, whose type strain was isolated from lake sediment (2). Genome sequence availability not only improves the resolution of *Clostridium* systematics but also provides deeper insights into their physiology and metabolism. Here, we present the draft genome sequences of five *Clostridium* strains. Strains WILCCON 0112 and WILCCON 0114 were isolated from dried and pelleted sunflower meal after oil extraction (obtained from Russia in November 2019) and from okara, a byproduct of soybean processing (obtained from China in August 2019), respectively. Strains WILCCON 0185, WILCCON 0202, and WILCCON 0269 were isolated from a soil sample collected with a sterile spatula from the topsoil layer below a cannonball tree (*Couroupita guianensis*) at Jurong Lake Gardens, Singapore (1.3359°N, 103.7262°E). For the isolation of strains WILCCON 0112 and WILCCON 0114, an aliquot of the sample was resuspended via vortexing and 10-fold serially diluted in phosphate-buffered saline (NaCl 137 mM, KCl 2.7 mM, Na_2_HPO_4_ 10 mM, and KH_2_PO_4_ 1.8 mM; pH 7.4) in four steps. A total of 100 µL of each dilution was spread on an MRS agar plate (1.6% agar) and incubated at 37°C under anoxic conditions (4% hydrogen, 5% carbon dioxide, and 91% nitrogen) for 48 h. Strains WILCCON 0185, WILCCON 0202, and WILCCON 0269 were obtained in a similar way but using ATCC 207, TS, or RCM agar plates, respectively, and incubating at 30°C. All strains underwent three rounds of purification on fresh agar plates.

Single colonies were inoculated into the respective liquid medium in 2 mL Eppendorf tubes and propagated stationary under the respective conditions as specified above. Nucleic acids were extracted from cell pellets using the Maxwell 16 FFS nucleic acid extraction system (Promega). Short-read, paired-end sequencing was conducted using the Illumina NovaSeq 6000 system (2 × 150 bp reads; NovogeneAIT Genomics Singapore) with DNA libraries prepared using the NEBNext Ultra DNA library preparation kit. The 10,231,748–11,875,844 raw reads generated were quality-trimmed using Trimmomatic version 0.39 (parameters: ILLUMINACLIP:*NovaSeq-adaptor-sequences.fa*:2:30:10 LEADING:3 TRAILING:3 SLIDINGWINDOW:4:15 MINLEN:36) (3). *De novo* assembly of the 10,091,090–11,765,546 clean reads obtained was performed using SPAdes version 3.15.5 (parameters: --careful --cov-cutoff auto; minimum contig length: 200 bp) (4). The resulting genome assemblies were evaluated for basic statistics using QUAST version 5.0.2 (5), assessed for completeness and contamination using CheckM version 1.2.2 (mode: lineage_wf) (6), and annotated using PGAP version 6.9 (default parameters) (7).

**Fig 1:**
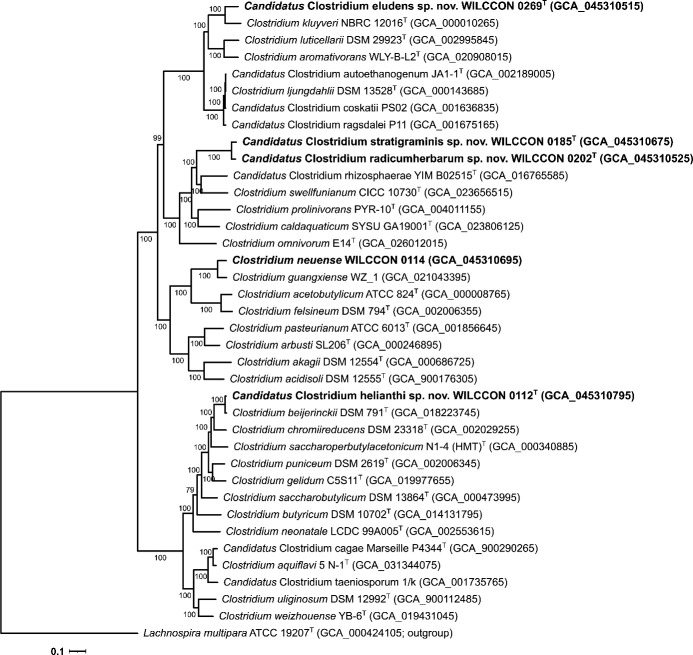
Phylogenomic tree showing the relationship between five *Clostridium* strains (in bold) and closely related taxa of the genus *Clostridium*. The maximum-likelihood tree was reconstructed as described previously (8, 9), based on the protein sequences of 361 single-copy core genes annotated using Prokka version 1.14.6 (default parameters) (10), identified and aligned using SCARAP version 0.4.0 (parameters: -p 100 -f 100) (11), and trimmed using trimAl version 1.4 (parameter: -automated1) (12). The tree was inferred with IQ-TREE version 2.1.2 (13) using the LG + F + I + G4 protein substitution model, rooted by midpoint-rooting, and visualized using Interactive Tree Of Life version 6.9.1 (14). *Lachnospira multipara* ATCC 19207^T^ from the neighboring family *Lachnospiraceae* was used as an outgroup. Ultrafast bootstrap values based on 1,000 replications are indicated at branching points. The scale bar denotes 0.1 substitutions per amino acid position.

Detailed assembly statistics and gene content information of the five draft genomes are summarized in Table 1. The taxonomic identities of the five *Clostridium* strains were validated by overall genomic relatedness indices and phylogenomic analyses (Table 1; Fig. 1). Strain WILCCON 0114 was assigned to *Clostridium neuense* based on 16S rRNA gene sequence similarity; this identification requires confirmation upon the availability of the type genome. The remaining four strains were proposed as *Candidatus* Clostridium species, namely, *Candidatus* Clostridium helianthi (WILCCON 0112), *Candidatus* Clostridium stratigraminis (WILCCON 0185), *Candidatus* Clostridium radicumherbarum (WILCCON 0202), and *Candidatus* Clostridium eludens (WILCCON 0269).

**TABLE 1:** Proposed taxonomic assignments and genome characteristics of five *Clostridium* strains^*a*^

Strain	WILCCON 0112	WILCCON 0114	WILCCON 0185	WILCCON 0202	WILCCON 0269
Isolation source	Sunflower meal	Okara	Soil	Soil	Soil
Taxonomic assignment	*Candidatus* Clostridium helianthi sp. nov.	*Clostridium neuense*	*Candidatus* Clostridium stratigraminis sp. nov.	*Candidatus* Clostridium radicumherbarum sp. nov.	*Candidatus* Clostridium eludens sp. nov.
Closest type strain	*Clostridium beijerinckii* DSM 791^T^	*Clostridium neuense* G1^T^	*Clostridium rhizosphaerae* YIM B02515^T^	*Clostridium rhizosphaerae* YIM B02515^T^	*Clostridium kluyveri* NBRC 12016^T^
16S rRNA GSS (%)	99.9	99.9	97.5	96.9	98.5
ANI (%)	96.2	NA	84.3	84.3	86.2
dDDH (%)	67.6	NA	20.1	20.8	28.7
BioProject acc. no.	PRJNA1187785	PRJNA1187786	PRJNA1187788	PRJNA1187789	PRJNA1187791
BioSample acc. no.	SAMN44804681	SAMN44804735	SAMN44804748	SAMN44804761	SAMN44804762
SRA acc. no. for raw reads	SRR31403109	SRR31393018	SRR31403192	SRR31403272	SRR31403754
No. of raw reads	11,427,098	11,875,844	10,488,504	10,231,748	10,825,438
Total bases of raw reads (bp)	1,714,064,700	1,781,376,600	1,573,275,600	1,534,762,200	1,623,815,700
No. of clean reads	11,302,930	11,765,546	10,406,918	10,091,090	10,731,566
Total bases of clean reads (bp)	1,681,815,945	1,751,505,498	1,552,151,258	1,501,551,961	1,599,543,825
WGS project acc. no.	JBJIAB000000000	JBJIAA000000000	JBJHZZ000000000	JBJHZY000000000	JBJHZX000000000
GenBank assembly acc. no.	GCA_045310795	GCA_045310695	GCA_045310675	GCA_045310525	GCA_045310515
Total genome size (bp)	6,059,073	5,021,087	3,657,445	4,053,182	5,093,834
Coverage	278×	349×	424×	370×	314×
DNA G + C content (%)	29.83	30.73	32.10	32.13	31.06
Completeness (%)	99.2	98.6	97.9	99.2	99.3
Contamination (%)	1.6	0.9	3.4	2.2	2.9
No. contigs	282	75	60	23	243
Largest contig	413,988	513,683	899,332	2,020,665	294,286
*N*_50_ (bp)	110,355	219,200	226,322	847,816	104,729
*L*_50_	16	9	4	2	17
Gene content					
No. coding sequences	5,474	4,626	3,474	3,859	5,118
No. tRNAs	72	64	71	72	64
No. ncRNAs	7	5	4	4	6
No. rRNAs	16	8	10	8	11
16S rRNA GenBank acc. no.	PQ625462	PQ614278	PQ625463	PQ625464	PQ625461
16S rRNA length (bp)	1,273	1,315	1,498	1,422	1,293

^*a*^ GenBank accession numbers (acc. no.) for 16S rRNA gene sequence and genome assembly of closely related type strains: *Clostridium beijerinckii* DSM 791^T^, X68179 and GCA_018223745; *Clostridium neuense* G1^T^, KT824779 and type genome not available; *Clostridium rhizosphaerae* YIM B02515^T^, MW911618 and GCA_016765585; *Clostridium kluyveri* NBRC 12016^T^, M59092 and GCA_000010265. Pairwise 16S rRNA gene sequence similarity (16S rRNA GSS), average nucleotide identity (ANI), and digital DNA-DNA hybridization (dDDH) prediction values were calculated using DSMZ single-gene phylogeny server (15), pyani version 0.2.11 (16), and Formula 2 of Genome-to-Genome Distance Calculator version 3.0 (17), respectively. 16S rRNA gene sequences were PCR-amplified using the 27F/1492R primer pair and GoTaq Master mix (Promega; settings: initial denaturation at 95°C for 2 min, followed by 30 cycles of annealing-extension at 95°C for 20 s, 55°C for 20 s and 72°C for 60 s, and final extension at 72°C for 8 min) and sequenced using the Sanger method.

## Data Availability

The 16S rRNA gene sequences of strains WILCCON 0112, WILCCON 0114, WILCCON 0185, WILCCON 0202, and WILCCON 0269 have been deposited in GenBank/ENA/DDBJ under accession numbers PQ625462, PQ614278, PQ625463, PQ625464, and PQ625461, respectively. The GenBank/ENA/DDBJ accession numbers for their whole genome shotgun project (WGS) are JBJIAB000000000, JBJIAA000000000, JBJHZZ000000000, JBJHZY000000000, and JBJHZX000000000, respectively. The Sequence Read Archive (SRA) accession numbers for their raw Illumina sequencing reads are SRR31403109, SRR31393018, SRR31403192, SRR31403272, and SRR31403754, respectively. The associated BioProject, BioSample, and genome assembly accession numbers are provided in Table 1.
